# An Important Movement

**Published:** 1880-07

**Authors:** 


### EDITORIAL.

### AN IMPORTANT MOVEMENT.

The following circular sufficiently explains the object of its issue. This movement should receive the careful and unbiased consideration of every member of the dental profession in this country who feels an interest or has taken an active part in association work.

The prime object of this movement is not to censure or reflect upon anything that has gone before; not to suplant or destroy anything that now exists; but to secure the co-operation and harmonious effort of a much larger number of the profession than hitherto, in one central body, whose suggestions and declarations shall be received, accepted and regarded as authority.

Let the aim be to secure the greatest good by associated effort; if the present organization is accomplishing this, than nothing further is needed, but if more and better can be done by a change, then let it come.

It is very desirable that this subject be thoroughly considered at the approaching meeting of the American Dental Association. The time has arrived when this subject cannot be ignored.

